# Extraction and restoration of hippocampal spatial memories with non-linear dynamical modeling

**DOI:** 10.3389/fnsys.2014.00097

**Published:** 2014-05-28

**Authors:** Dong Song, Madhuri Harway, Vasilis Z. Marmarelis, Robert E. Hampson, Sam A. Deadwyler, Theodore W. Berger

**Affiliations:** ^1^Department of Biomedical Engineering, University of Southern CaliforniaLos Angeles, CA, USA; ^2^Department of Physiology and Pharmacology, School of Medicine, Wake Forest UniversityWinston-Salem, NC, USA

**Keywords:** hippocampus, spatio-temporal pattern, spike, classification, regression, memory

## Abstract

To build a cognitive prosthesis that can replace the memory function of the hippocampus, it is essential to model the input-output function of the damaged hippocampal region, so the prosthetic device can stimulate the downstream hippocampal region, e.g., CA1, with the output signal, e.g., CA1 spike trains, predicted from the ongoing input signal, e.g., CA3 spike trains, and the identified input-output function, e.g., CA3-CA1 model. In order for the downstream region to form appropriate long-term memories based on the restored output signal, furthermore, the output signal should contain sufficient information about the memories that the animal has formed. In this study, we verify this premise by applying regression and classification modelings of the spatio-temporal patterns of spike trains to the hippocampal CA3 and CA1 data recorded from rats performing a memory-dependent delayed non-match-to-sample (DNMS) task. The regression model is essentially the multiple-input, multiple-output (MIMO) non-linear dynamical model of spike train transformation. It predicts the output spike trains based on the input spike trains and thus restores the output signal. In addition, the classification model interprets the signal by relating the spatio-temporal patterns to the memory events. We have found that: (1) both hippocampal CA3 and CA1 spike trains contain sufficient information for predicting the locations of the sample responses (i.e., left and right memories) during the DNMS task; and more importantly (2) the CA1 spike trains predicted from the CA3 spike trains by the MIMO model also are sufficient for predicting the locations on a single-trial basis. These results show quantitatively that, with a moderate number of unitary recordings from the hippocampus, the MIMO non-linear dynamical model is able to extract and restore spatial memory information for the formation of long-term memories and thus can serve as the computational basis of the hippocampal memory prosthesis.

## Introduction

**Figure 1:**
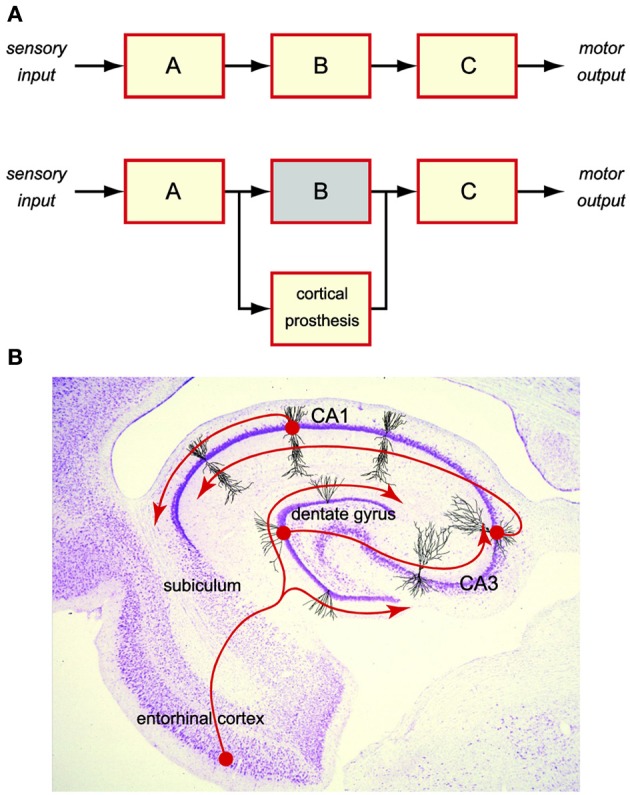
**Cortical prosthesis restores cognitive functions by bypassing the damaged brain region to maintain the brain signal flow**. **(A)** Schematic diagram of a cortical prosthesis. **(B)** Intrinsic tri-synaptic pathway of the hippocampus.

Cortical prosthesis is an emerging technology seeking to restore cognitive functions lost in diseases or injuries (Berger et al., 2005, 2010, 2011, 2012). It is achieved by bi-directional, closed-loop communications between the prosthetic device and the brain regions. This is distinct from sensory or motor prostheses, where one side of the communication is an external entity such as the sensory input (Loeb, 1990; Humayun et al., 1999) or the motor output (Mauritz and Peckham, 1987; Taylor et al., 2002; Nicolelis, 2003; Shenoy et al., 2003; Wolpaw and McFarland, 2004; Hochberg et al., 2006). Therefore, a cortical prosthesis must deal exclusively with the internal brain signals, in which sensory or motor information is embedded, by re-encoding the upstream (input) brain signals into the downstream (output) signals (Figure 1A).

For the past decade, we have been working on developing a hippocampal-cortical prosthesis for restoring the memory functions. Hippocampus is a brain region responsible for the creation of new long-term episodic memories (Milner, 1970; Squire and Zola-Morgan, 1991; Eichenbaum, 1999). Damage to the hippocampal areas can result in a permanent loss of such cognitive functions. In a normal hippocampus, short-term memories are encoded in the spatio-temporal patterns of spikes (i.e., spike trains) as the input from the entorhinal cortex. Memory information is then processed by the hippocampal feedforward tri-synaptic pathway, which consists of dentate gyrus, CA3, and CA1 regions, and eventually transformed into the output spike trains to the subiculum, that is appropriate for the formation of long-term memories (Figure 1B). Although the exact nature of such a transformation or the underlying mechanisms is still largely unclear, it must be the neural signal (i.e., spike trains) flow from entorhinal cortex to dentate gyrus, to CA3, to CA1, and to subiculum, that enables the re-encoding of short-term memories into long-term memories. Maintaining the normal signal flow with a prosthetic device that bypasses a damaged or diseased hippocampal region provides a feasible way of restoring the lost long-term memory functions (Figure 1A).

For example, in our first-generation hippocampal memory prosthesis applications, we (a) record input spike trains from the CA3 region, (b) process them with a multi-input, multi-output (MIMO) non-linear dynamical model to predict the desired CA1 output spike trains, and (c) electrically stimulate the CA1 region with the predicted CA1 output patterns. Previous results have shown that, (a) the MIMO model can accurately predict the output spike trains in real time based on the ongoing input spike trains (Song et al., 2007, 2009a, 2013), and (b) the electrical stimulation can restore or even enhance the memory functions performed by the hippocampal CA3-CA1 system (Berger et al., 2011, 2012; Hampson et al., 2012a,b).

**Figure 2:**
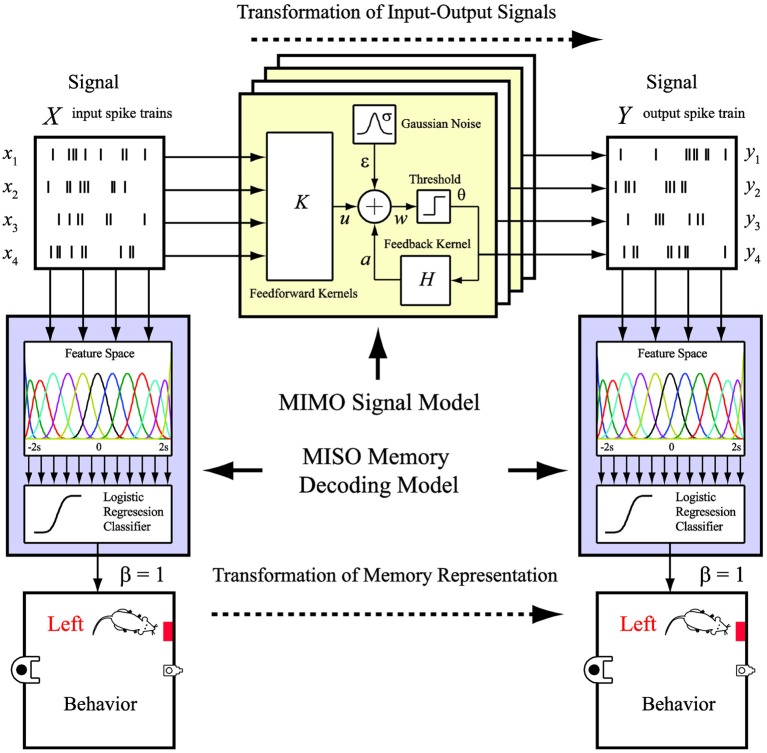
**MIMO signal model and MISO memory decoding model**. The MIMO signal model predicts the output spatio-temporal patterns of spikes based on the ongoing input spatio-temporal patterns of spikes. The MISO memory decoding model predicts the memory (behavioral) events based on the input or output spatio-temporal patterns of spikes.

However, despite the success of demonstrating such a prosthesis, how the external behavioral events (i.e., memory events) are encoded in the two hippocampal regions and, more importantly, re-encoded by the prosthesis has not been clearly revealed, precisely due to the internal nature of the cortical prosthesis. In this study, we propose a new framework of modeling and representing the re-encoding process performed by a brain region at the *memory representation level*, as opposed to the *signal level* in our previous studies. In addition to ask the question, “What should the output signal be?” We further ask the question, “What do the signals mean?” Specifically, we combine our previously developed MIMO signal model (Song et al., 2007, 2009a, 2013), which predicts the output signal based on the input signal, with an additional memory decoding model that relates the input and/or output signals to the behaviors (memories) of the animal (Figure 2). The MIMO signal model is essentially a time-series regression model non-linear dynamically mapping the multiple output (CA1) signals to the multiple input (CA3) signals. On the other hand, the memory decoding model is a multi-input, signal-output (MISO) classification model identifying to which of a set of memory categories the spatio-temporal patterns of the input and/or output signals belong. The former model quantifies the input-output signal transformation, while the latter model decodes the memory by predicting the behavior.

The paper is organized as the follows. In section Materials and Methods, we formally formulate the modeling problem and provide the mathematical expressions. In section Results, we apply the methods to the modeling of the hippocampus during a memory-dependent task in rodents.

## Materials and methods

### Behavioral task and electrophysiological procedures

**Figure 3:**
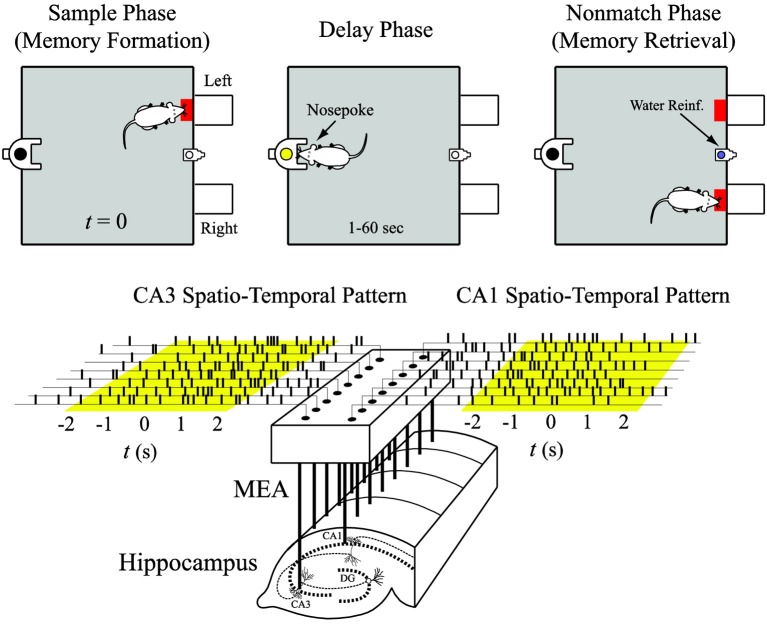
**Input (CA3) and output (CA1) spatio-temporal patterns of spikes recorded with a multi-electrode array (MEA) during the sample phase of the delayed-non-match-to-sample (DNMS) task**.

All animal procedures are reviewed and approved by the Institutional Animal Care and Use Committee of Wake Forest University, in accordance with US Department of Agriculture, International Association for the Assessment and Accreditation of Laboratory Animal Care and National Institutes of Health guidelines. Two male Long-Evans rats are trained to criterion on a two-lever, spatial delayed-non-match-to-sample (DNMS) task with random delay intervals (Deadwyler et al., 1996; Hampson et al., 1999). Animals perform the task by pressing (sample response) a single lever presented in one of the two positions in the sample phase (left or right). This event is called the “sample response.” The lever is then retracted and the delay phase initiates; for the duration of the delay phase, the animal is required to nose-poke into a lighted device on the opposite wall. When the delay is ended, nose-poke light is extinguished, both levers are extended, and the animal is required to press the lever *opposite* to the sample lever. This event is called the “non-match response.” If the correct lever is pressed, the animal is rewarded (Figure 3, top). A session includes approximately 100 successful DNMS tasks that each consists of two of the four behavioral events, i.e., right sample (RS) and left non-match (LN), or left sample (LS) and right non-match (RN).

Spike trains are obtained with multi-site recordings from different septo-temporal regions of the hippocampus of rats performing the DNMS task (Figure 3, bottom). For each hemisphere of the brain, a microwire multi-electrodes array (MEA) is surgically implanted into the hippocampus, with 8 electrodes in the CA3 (input) region and 8 electrodes in the CA1 (output) region. Spike trains are pre-screened based on mean firing rate and peri-event histogram. Perievent (−2 to +2 s) spike trains of the four behavioral events are extracted from each trial and then concatenated to form the datasets (Figure 3, bottom). The spike train data are discretized with a 2 ms bin size.

### MIMO signal model of input-output spike train transformation

The MIMO signal model of input-output spike train transformation takes the form of the sparse generalized Laguerre-Volterra model (SGLVM) we previously developed (Song et al., 2009a,b, 2013). In this approach, a MIMO model is a concatenation of a series of MISO models (not to be confused with the MISO classification model), that each can be considered a spiking neuron model (Song et al., 2006, 2007) (Figure 2). In this study, each MISO model consists of (a) MISO second-order Volterra kernels *k* transforming the input spike trains *x* to the synaptic potential *u*, (b) a Gaussian noise term ε capturing the stochastic properties of spike generation, (c) a threshold θ for generating output spikes *y*, (e) an adder generating the pre-threshold membrane potential *w*, and (d) a single-input, single-output first-order Volterra kernel *h* transforming the preceding output spikes to the spike-triggered feedback after-potential *a*. The model can be mathematical expressed as:

(1) w=u(k,x)+a(h,y)+ε(σ)

(2) $$y=\{\begin{array}{cc} 0 & \text{when }w<\theta \\ 1 & \text{when }w\geq \theta \end{array}$$

(3) $$\begin{array}{l} u(t)=k_{0}+\sum_{n\;=\;1}^{N}\sum_{\tau \;=\;0}^{M_{k}}k_{1}^{\left(n\right)}(\tau )x_{n}(t-\tau )\\ \;+\sum_{n\;=\;1}^{N}\sum_{\tau_{1}\;=\;0}^{M_{k}}\sum_{\tau_{2}\;=\;0}^{M_{k}}k_{2s}^{\left(n\right)}(\tau_{1},\tau_{2})x_{n}(t-\tau_{1})x_{n}(t-\tau_{2}) \end{array}$$

(4) $$a(t)=\sum_{\tau \;=\;1}^{M_{h}}h(\tau )y(t-\tau )$$

The zeroth-order kernel, *k*_0_, is the value of *u* when the input is absent. First-order kernels *k*^(*n*)^_1_ describe the first-order linear relation between the *n*^*th*^ input *x*_*n*_ and *u*, as functions of the time intervals τ between the present time and the past time. Second-order self kernels *k*^(*n*)^_2*s*_ describe the second-order non-linear interaction between pairs of spikes in the *n*^*th*^ input *x*_*n*_ as they affect *u*. *N* is the number of inputs. *M*_*k*_ and *M*_*h*_ denote the memory lengths of the feedforward process and feedback process, respectively. They are chosen to be 2 s in this study. Second-order cross kernels and higher-order (e.g., third-order) kernels are not included in this study.

To facilitate model estimation and avoid overfitting, the Volterra kernels are expanded with Laguerre basis functions *b* as in Song et al. (2009c,d):

(5) $$\begin{array}{l} u(t)=c_{0}+\sum_{n\;=\;1}^{N}\sum_{j\;=\;1}^{J}c_{1}^{\left(n\right)}(j)v_{j}^{\left(n\right)}(t)\\ \;+\sum_{n\;=\;1}^{N}\sum_{j_{1}\;=\;1}^{J}\sum_{j_{2}\;=\;1}^{j_{1}}c_{2s}^{\left(n\right)}(j_{1},j_{2})v_{j_{1}}^{\left(n\right)}(t)v_{j_{2}}^{\left(n\right)}(t) \end{array}$$

(6) $$a(t)=\sum_{j\;=\;1}^{L}c_{h}(j)v_{j}^{\left(h\right)}(t)$$

where $v_{j}^{\left(n\right)}(t)=\sum_{\tau \;=\;0}^{M_{k}}b_{j}(\tau )x_{n}(t-\tau )$, $v_{j}^{\left(h\right)}(t)=\sum_{\tau \;=\;1}^{M_{h}}b_{j}(\tau )$ *y*(*t* − τ); *c*^(*n*)^_1_, *c*^(*n*)^_2*s*_, and *c*_*h*_ are the sought Laguerre expansion coefficients of *k*^(*n*)^_1_, *k*^(*n*)^_2*s*_, and *h*, respectively (*c*_0_ is equal to *k*_0_); *J* is the number of basis functions.

To achieve model sparsity, the coefficients are estimated with a composite penalized likelihood estimation method, i.e., group LASSO (Song et al., 2013). In maximum likelihood estimation (MLE), model coefficients are estimated by minimizing the negative log likelihood function -*l*(*c*). In group LASSO, the composite penalized criterion is written as

(7) $$\begin{array}{l} S(c)=-l(c)+\lambda \text{​}\left(\sum_{n\;=\;1}^{N}||c_{1}^{\left(n\right)}(j)|{|}_{2}^{1}+\sum_{n\;=\;1}^{N}||c_{2s}^{\left(n\right)}(j_{1},j_{2})|{|}_{2}^{1}\right)\\ \;=-l(c)+\lambda \text{​}(\sum_{n\;=\;1}^{N}{\left(\sum_{j\;=\;1}^{J}c_{1}^{\left(n\right)}{\left(j\right)}^{2}\right)}^{\frac{1}{2}}\\ \;+\sum_{n\;=\;1}^{N}{\left(\sum_{j_{1}\;=\;1}^{J}\sum_{j_{2}\;=\;1}^{j_{1}}c^{\left(n\right)}{\left(j_{1},j_{2}\right)}^{2}\right)}^{\frac{1}{2}}) \end{array}$$

where λ ≥ 0 is a tuning parameter that controls the relative importance of the likelihood and the penalty term. When λ takes on a larger value, the estimation yields sparser result of the coefficients. λ is optimized with a two-fold cross-validation method.

### MISO memory decoding model of spatio-temporal pattern of spikes

The MISO memory decoding model of spike spatio-temporal patterns takes the form of the sparse generalized B-spline linear classification model (Song et al., 2013). In this approach, the feature space is defined as a set of B-spline basis functions for each neuron (input and/or output neurons depending on the application). The classifier is essentially the logistic regression (Figure 2).

B-splines are piecewise polynomials with smooth transitions between the adjacent pieces at a set of interior *knot* points. A polynomial spline of degree *d* ≥ 0 on [0, *M*] with *m* > 0 interior knot points and the knot sequence η_0_ = 0 < η_1_ < … < η_*m*_ < η_*m* + 1_ = *M* is a function that is a polynomial of degree *d* between each pair of adjacent knots, and has *d*-1 continuous derivatives for *d* = 1. B-spline basis functions of degree *d* can be defined in a recursive fashion as

(8) $$B_{j,d}(\tau )=\frac{\tau -\eta_{j}}{\eta_{j\;+\;d\;-\;1}-\eta_{j}}B_{j,d-1}(\tau )+\frac{\eta_{j\;+\;d}-\tau }{\eta_{j\;+\;d}-\eta_{j\;+\;1}}B_{j\;+\;1,d-1}(\tau )$$

where

(9) $$B_{j,0}(\tau )=\{\begin{array}{ll} 1 & \text{ if }\eta_{\text{j}}<\tau <\eta_{j\;+\;1}\\ 0 & \text{ otherwise} \end{array}$$

For a given sequence of *m* knots and a fixed degree *d*, the total number of B-spline basis functions is *J* = *m* + *d* + 1.

Spatio-temporal patterns of spikes are projected to the B-spline feature space via inner product to yield the feature vectors as

(10) $$z^{\left(n\right)}(j)=\sum_{\tau \;=\;0}^{M}B_{j}(\tau )x_{n}(\tau )$$

where *M* is the time widow for inner product. It is chosen to be from −2 to +2 s of the sample events (Figure 3, bottom). *x*_*n*_ is the *n*th neuron of the total *N* neurons included in analysis. Different from in the regression model, *x* can be CA3 and/or CA1 neurons depending on the context. *z*^(*n*)^(*j*) denotes the feature value of the *n*th neuron using the *j*th B-spline function. Therefore, *z* is a 1-by-*JN* vector. *J* is optimized in the range of 5–100 based on the out-of-sample prediction accuracy. In most of the cases, *J* = 20 is found to be optimal.

Since there are two possible behavioral outcomes, i.e., left or right position, the model output can be represented as a binary variable β. The classification model assumed by logistic regression is

(11) $$P(\beta =1|x)={\left[1+\exp\left\{-w_{0}-\sum_{n\;=\;1}^{N}\sum_{j\;=\;1}^{J}w^{\left(n\right)}(j)z^{\left(n\right)}(j)\right\}\right]}^{-1}$$

(12) P(β=0|x)=1−P(β=1|x)

where *w* are the sought model coefficients; 1 and 0 represent left and right positions, respectively.

The linear classification rule is simply

(13) $$\beta =\{\begin{array}{ll} 1 & \text{ if }\left\{-w_{0}-\sum_{n\;=\;1}^{N}\sum_{j\;=\;1}^{J}w^{\left(n\right)}(j)z^{\left(n\right)}(j)\right\}<0\\ 0 & \text{ otherwise} \end{array}$$

Compared with the MIMO regression model, the MISO classification model may suffer even more serious overfitting problem due to the high dimensional input (typically with hundreds of features) and the relatively small number of data points (typically 100 trials in this study). Therefore, *L*1 regularization (Lasso) is applied to achieve model sparsity and avoid overfitting as

(14) $$S(c)=-l(c)+\lambda \left(\sum_{n\;=\;1}^{N}\sum_{j\;=\;1}^{J}||w^{\left(n\right)}(j)|{|}_{2}^{1}\right)$$

Where −*l*(*c*) and λ = 0 are the negative log likelihood function and the tuning parameter of the classification model, respectively. In this study, λ is optimized with a four-fold cross-validation method. By minimizing *S*, sparse weight matrix *w* are estimated and further used to reconstruct the classification feature matrix *F* with the B-spline basis functions as

(15) $$F^{\left(n\right)}(\tau )=\sum_{j\;=\;1}^{J}B_{j}(\tau )w^{\left(n\right)}(j)$$

*F* can be directly used in the logistic regression along with the spatio-temporal pattern *x* as

(16) $$P(\beta =1|x)={\left[1+\exp\left\{-w_{0}-\sum_{n\;=\;1}^{N}\sum_{t\;=\;1}^{M}F^{\left(n\right)}(\tau )x^{\left(n\right)}(t)\right\}\right]}^{-1}$$

## Results

### Hippocampal CA3 and CA1 activities contains sufficient information for decoding spatial memories during the DNMS task

**Figure 4:**
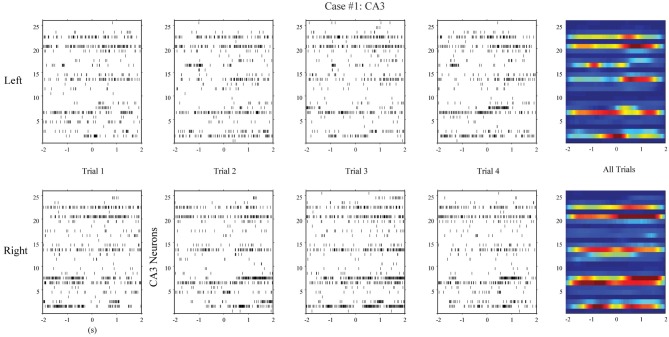
**Spatio-temporal patterns of spikes in the hippocampal CA3 (input) region during left and right sample events of the DNMS (case #1)**.

**Figure 5:**
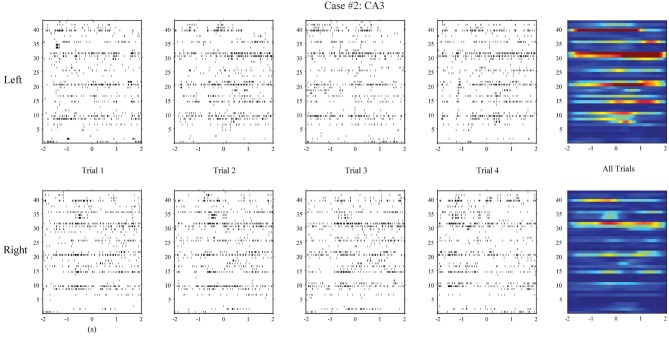
**Hippocampal CA3 spatio-temporal patterns from a different animal (case #2)**.

First, we apply the MISO memory decoding model to the CA3 spike trains recorded during the sample phase of the DNMS tasks. For each sample event (left or right), we take the perievent spikes 2 s before and after the event with a 2 ms bin size. The spatio-temporal patterns of spikes are then *N*-by-2000 matrices, where *N* is the number of neurons. A session typically consists of 80–100 trials with roughly half being left sample trials and half being right sample trials. The spatio-temporal patterns are labeled with 1 for the left trials and 0 for the right trials. Figure 4 (case #1) and Figure 5 (case #2) show the spatio-temporal patterns from two animals with 26 and 43 CA3 neurons, respectively. For each position, four representative patterns and the overall patterns are shown. The overall patterns are obtained by smoothing the spike trains with B-spline functions and then summing across all trials for the specific position. It is evident that the two positions show different spatio-temporal patterns and the differences exist in specific time ranges of specific neurons (Figures 4, 5). The task of the MISO memory decoding model is to identify these sparsely distributed differences from single trials of the spatio-temporal pattern and then predict the positions of the animal. Results show that the MISO memory decoding model can achieve a 100% out-of-sample prediction accuracy using the CA3 spatio-temporal patterns in both cases (Figure 8, top row).

**Figure 6:**
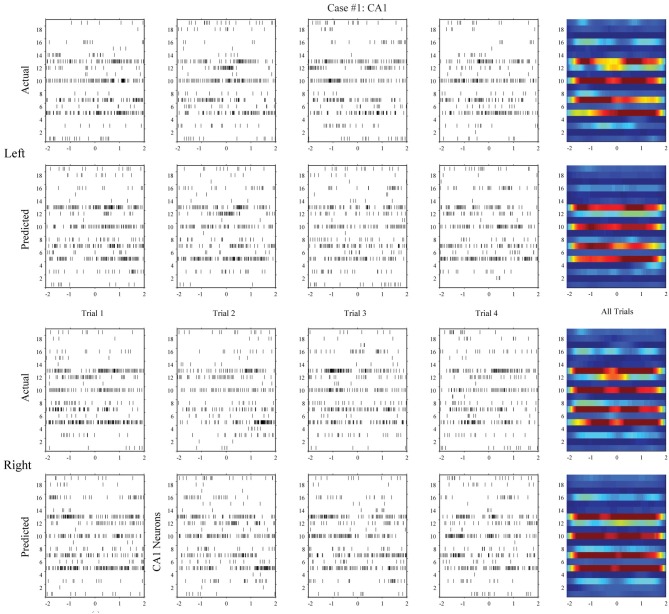
**Spatio-temporal patterns of spikes in the hippocampal CA1 (output) region during left and right events of the DNMS**. For each position, the top row shows the actual CA1 spatio-temporal patterns; the bottom row shows the CA1 spatio-temporal patterns predicted by the MIMO model based on the ongoing CA3 spiking activities (case #1).

**Figure 7:**
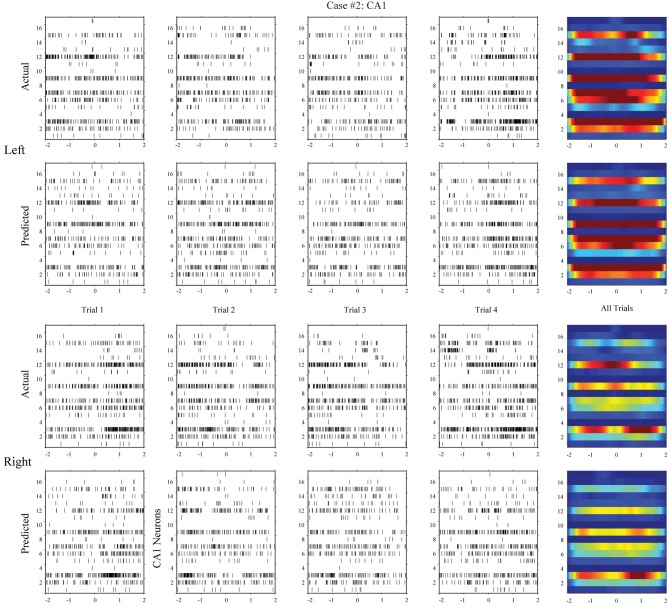
**Hippocampal CA1 spatio-temporal patterns (actual and MIMO model predicted) from a different animal (case #2)**.

**Figure 8:**
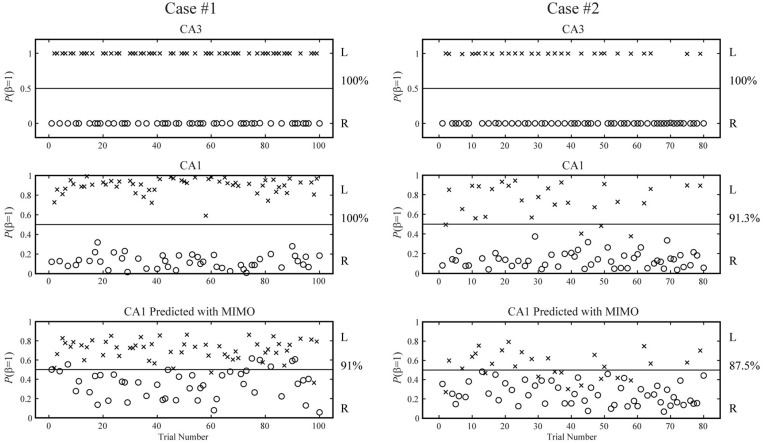
**Predicting spatial memory events based on inputs (CA3), outputs (CA1), or outputs predicted by the MIMO models (case #1 and #2)**. The horizontal lines in the middle represent the decision boundaries (*P* = 0.5).

Using the same method, we build MISO memory decoding models for the CA1 spike trains. Figure 6 (case #1) and Figure 7 (case #2) show the spatio-temporal patterns of CA1 during left and right trials (row 1 and 3) from the same two animals. There are 19 and 17 CA1 neurons recorded from these two animals, respectively. Similar to CA3, CA1 also show different spatio-temporal patterns during left and right trials. The prediction accuracy is 100% in one case and 91.3% in the other (Figure 8, middle row).

### Hippocampal CA1 activities can be accurately predicted by the MIMO signal model based on the hippocampal CA3 activities

In the second step, we build MIMO signal models for the transformations from the CA3 spatio-temporal patterns to the CA1 spatio-temporal patterns. To build such a model, we concatenate CA3 perievent spike trains across all trials to form the input data and the corresponding CA1 spike trains to form the output data, and then apply our MIMO modeling method. The resulting SGLVM non-linear dynamically predicts the CA1 spikes based on the ongoing and past (within the memory window) CA3 spikes (Song et al., 2007, 2009a; Song and Berger, 2010). Results show that in both cases (Figures 6, 7, row 2 and 4), the MIMO signal model can accurately predict the CA1 spatio-temporal patterns at both the single trial level (Figures 6, 7, column 1–4) and the overall level (Figures 6, 7, column 5). Importantly, a single set of the model coefficients are used for both the left and right trials. In other words, the estimated MIMO signal models are memory-invariant and can be used to predict the output signals without explicitly knowing what the events are (Song et al., 2011).

### Hippocampal CA1 activities predicted by the MIMO signal model can be used to accurately decode the spatial memory

Lastly, we build MISO memory decoding models for the CA1 spatio-temporal patterns predicted by the MIMO signal model, as opposed to the actual CA1 spatio-temporal patterns. Results show that the MISO memory decoding models can accurately predict the spatial memory based on the predicted CA1 patterns (Figure 8, bottom). The prediction accuracies are 91% and 87.5 for the two cases, respectively. Importantly, the MISO memory decoding model coefficients remain the same for the actual CA1 patterns and the predicted CA1 patterns. This indicates that the MIMO signal model has successfully transmitted the spatial information from CA3 to CA1 in the same form as it is encoded in the actual CA1 patterns. The MIMO signal model has not only *restored the signal*, but also *re-encoded the memory representations*.

### Spatial information is sparsely distributed in the hippocampal CA1 and CA3 spatio-temporal patterns of spikes

**Figure 9:**
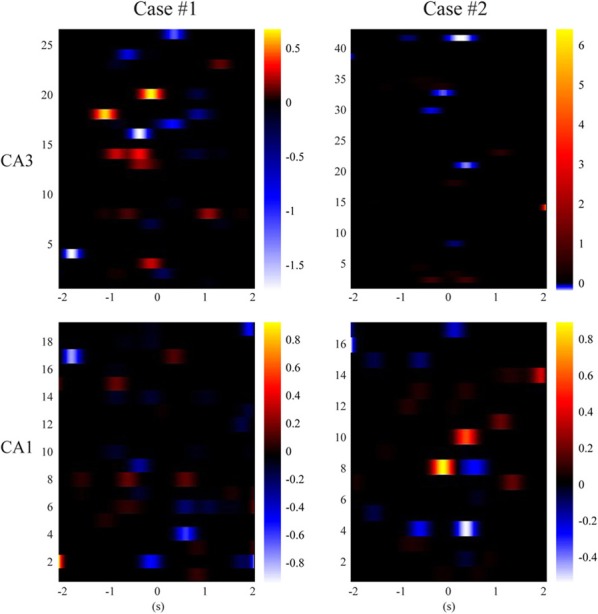
**Sparse classification feature matrices of the MISO memory decoding models (case #1 and #2)**. Black color represents zero-valued weights.

In order to gain more insights into how hippocampal CA3 and CA1 spike trains encode spatial information, we calculate Equation (15) and plot the classification weight matrices. These matrices have the same dimensions as their corresponding spatio-temporal patterns. In order to perform classification, we can simply calculate the dot products of the weight matrices and the corresponding spatio-temporal patterns (strictly speaking, the dot products of vectorized matrices), add the bias (i.e., *w*_0_), and then use Equation (16) to predict the probability of the animal having left or right memories. Figure 9 show results of CA3 and CA1 from the two animals. The CA1 weight matrices are for both the actual and MIMO predicted CA1 spatio-temporal patterns. In both cases, non-zero values (warm and cold colors represent positive and negative values, respectively) are sparsely distributed in the weight matrices. These results indicate that the spatial information exists in a redundant fashion in multiple ranges of the perievent intervals of multiple neurons. The MIMO signal model and the MISO memory decoding model jointly describe the re-encoding of the memory representations from CA3 to CA1.

## Discussions

Brain regions process and transmit information with spatio-temporal patterns of spikes. In order to build a cortical prosthesis to bypass a damaged brain region, it is necessary to restore the output signals of the damaged region and send it to the downstream region, so the information flow is maintained. We have shown intensively that non-linear dynamical MIMO models can predict accurately the output spatio-temporal patterns based on the ongoing input spatio-temporal patterns, and electrical stimulations of the output region following the predicted patterns can effectively restore and even enhance the memory function (Berger et al., 2011, 2012; Hampson et al., 2012a,b, 2013). The unique contribution of this paper is to combine the MIMO models with a new set of MISO memory decoding models so that the input and output signals can be related to the memory (behavioral) events and thus explain why it is possible for the downstream hippocampal region to correctly decode the MIMO model generated signals.

In our previous publications on the MIMO signal model (Song et al., 2006, 2007, 2009a,b, 2011, 2013; Song and Berger, 2010), the model goodness-of-fit are validated with a Kolmogorov-Smirnov (KS) test based on the time-rescaling theorem (Brown et al., 2002; Haslinger et al., 2010). This KS test is a powerful tool that allows the firing probability intensity function predicted by the MIMO model to be directly validated with the actual output spike train, and the model goodness-of-fit to be quantified statistically with confidence bounds. However, the KS test does not necessarily indicate whether the model goodness-of-fit is sufficient for decoding the behavior or restoring the cognitive function since it is developed only for quantifying the accuracy of the predicted point-process output signal. The typically used 95 or 99% confidence bounds will not guarantee a successful MIMO model for building the prosthesis. For example, a perfectly predicted output signal may contain no information about a specific memory of interest; on the other hand, a less accurately predicted output signal may still contain some or even sufficient information about the memory. The MISO memory decoding model described here directly quantifies the relations between output signals and memories, and provides a more functionally relevant measure to the model performance that is complementary to the KS test.

In hippocampal prosthesis applications, MISO memory decoding models are estimated with input-output data during the sample phase (−2 to 2 s). The reason is that, in the DNMS task, animals form the spatial memory (i.e., left or right level position) during the sample phase, retain the memory during the delay phase, and recall the memory during the non-match phase. Previous results have shown that MIMO model-based electrical stimulation restores and enhances the spatial memory during sample phases but not non-match phases (Berger et al., 2011, 2012; Hampson et al., 2012a), despite that the MIMO signal model is able to predict accurately the output signal during both sample and non-match phases (Song et al., 2011).

Hippocampus is a mainly feedforward network consisting of a large number of neurons. There are approximately 1 million, 330 thousand, and 420 thousand principal neurons in the rodent dentate gyrus, CA3, and CA1 regions, respectively (Amaral et al., 1990). However, despite the small number (tens to a hundred) of recorded neurons allowed by the current MEA technology, our hippocampal prosthesis has shown impressive success in both rodents and non-human primates during the spatial memory tasks. The main reason is that, at least during the DNMS task or the delayed match-to-sample (DMS) task, spatial memories (e.g., locations of the levels) are encoded in a highly redundant and distributed fashion in a large portion of the hippocampal neurons. As shown in this study, sampling a small number of neurons from the whole population still allows accurate extraction of spatial information.

The DNMS task is a highly restricted experimental paradigm that involves only two positions. Under normal conditions, however, the animal needs to form much more complex memories to maintain its normal life (Eichenbaum, 1999). A practical hippocampal prosthesis should be able to extract and restore a large number of memories with the MIMO signal model and MISO memory decoding model. This will likely require (1) recording a larger number of hippocampal neurons to obtain more information necessary for decoding the episodic memories, (2) stimulating with more electrodes for generating richer output patterns to the downstream hippocampal region, and (3) developing more powerful MIMO signal model and MISO memory decoding model to more accurately restore the output signal and decode the memories. For example, the current MISO memory decoding model has binary (left or right) output; in order to decode more memories, it needs to be extended to handle multiple-category output. A natural solution is to use multinomial logistic regression (McCullagh and Nelder, 1989), instead of the standard binary-output logistic regression used in this study. Besides, other forms of discriminative models, e.g., support vector machine, or generative models, e.g., naive Bayes classifier, may be considered for their specific advantages. In addition, to collect input-output data for building multiple-memory models, new experimental paradigms involving multiple forms of behavioral events and sensory modalities need to be utilized (Hampson et al., 2012b). Nonetheless, the study described in the paper for the first time combines the regression model with the classification model to illustrate how memory-related information is encoded and re-encoded in the hippocampus, and has made a critical step toward building a hippocampal memory prosthesis.

Interestingly, in both cases in this study, the MISO memory decoding model shows higher prediction accuracy in the CA3 than in the actual CA1, and higher accuracy in the actual CA1 than in the predicted CA1. The latter is unsurprising since the predicted CA1 patterns are calculated with the MIMO signal model using the actual CA1 patterns as target signals, although the real-time calculation is driven by the ongoing CA3 patterns. It is thus unlikely for the predicted CA1 patterns to contain more memory-related information than the actual CA1 patterns. The former observation can be caused by two factors. First, it is possible that CA3 neurons contain more spatial information than CA1 neurons as suggested by previous studies (Lee et al., 2004). Second, it could simply due to the fact that we have recorded more CA3 units than CA1 units in the two cases included in this study. A more systematic, comparative study of CA3 and CA1 patterns needs to be performed to draw further conclusions.

In this study, the MISO memory decoding model takes the form of a B-spline, logistic regression model. The B-spline basis functions are utilized to reduce the model dimensionality and introduce a continuous metric for the similarities between spike trains. The optimal number of basis functions provides an estimate to the relevant temporal resolution of the spike trains. The logistic regression maps the spatio-temporal features to the probability of having a certain behavioral outcome. Despite the rather general model structure and the high prediction accuracy, however, this study does not necessarily suggest that the downstream hippocampal region decodes the CA1 spatio-temporal patterns in the same way. Instead, the main biological implications of this study are: first, the CA1 spatio-temporal patterns can be accurately predicted from the CA3 spatio-temporal patterns using a non-linear dynamical MIMO signal model; second, both CA3 and CA1 patterns contains sufficient information for decoding the memory events; third, the MIMO-model predicted CA1 patterns also contain sufficient information of the memory, and it must be this fact that makes the successful implementation of the hippocampal memory prostheses possible.

### Conflict of interest statement

The authors declare that the research was conducted in the absence of any commercial or financial relationships that could be construed as a potential conflict of interest.
